# Vector competence of *Aedes albopictus* and *Culex quinquefasciatus* for Oropouche virus

**DOI:** 10.1371/journal.pntd.0014656

**Published:** 2026-10-23

**Authors:** Angel Elma I. Abu, Xinyi Hua, Rachel Sattler, Margaret V. Becker, Julia Forato, Shirlene T. S. de Lima, Ingra M. Claro, Gabriel C. Scachetti, Leda M. Simões Mello, Maria Anice M. Sallum, Kenneth S. Plante, Scott C. Weaver, José Luiz Proenca-Modena, William M. de Souza, Laura B. Dickson

**Affiliations:** 1 Department of Microbiology and Immunology, University of Texas Medical Branch, Galveston, Texas, United States of America; 2 Department of Microbiology, Immunology, and Molecular Genetics, College of Medicine, University of Kentucky, Lexington, Kentucky, United States of America; 3 World Reference Center for Emerging Viruses and Arboviruses, University of Texas Medical Branch, Galveston, Texas, United States of America; 4 Laboratory of Emerging Viruses, Department of Genetics, Microbiology and Immunology, Institute of Biology, University of Campinas, Campinas, Brazil; 5 Departamento de Epidemiologia, Faculdade de Saúde Pública, Universidade de São Paulo, São Paulo, Brazil; Connecticut Agricultural Experiment Station, UNITED STATES OF AMERICA

## Abstract

Oropouche virus (OROV) is an arthropod-borne virus transmitted mainly by *Culicoides paraensis* midges in Latin America and the Caribbean, though evidence suggests occasional transmission by mosquitoes. Oropouche fever has been reported primarily in the Amazon Basin since the 1950s. In 2024, the number of Oropouche fever cases rose sharply and spread beyond the Amazon region, which has been associated with the emergence of a new reassortant OROV strain. We assessed whether increased case numbers could be explained by enhanced replication or transmission of the reassortant strain in peri-urban *Culex quinquefasciatus* and *Aedes albopictus*. Laboratory colonies established from Salvador, Brazil were orally exposed to the ancestral OROV strain BeAn 19991 or the contemporary strain 240023 (10⁴–10⁶ PFU/mL), and viral replication was quantified in bodies, legs, and saliva. To evaluate the influence of mosquito genotype on infection outcomes, infection rates were compared between *Ae. albopictus* from Salvador City (Brazil) and Houston (USA). In *Cx. quinquefasciatus*, the outbreak strain replicated less efficiently than the historical strain at low viral doses but not at high infection doses. Regardless of strain or dose, vector competence was extremely low, with only 0–1% of mosquitoes exhibiting infectious OROV particles in their saliva after extrinsic incubation. Conversely, *Ae. albopictus* showed consistently higher body infection rates for the 240023 strain than for BeAn 19991 at both low and high doses. However, overall transmission potential remained limited: only 0–6.4% of leg or saliva samples were positive across OROV strains and doses. The higher infection rates of the 240023 strain was consistent across both *Ae. albopictus* genotypes, but infectious saliva was detected only in mosquitoes from the Salvador line (up to 20%) and not in the Houston line (0%). Taken together, this suggests that the recent geographic expansion of OROV is unlikely to be driven by a vector shift from midges to these mosquitoes. Importantly, our findings do indicate higher infectivity of the 240023 strain in *Ae. albopictus* across different mosquito lines which may have future implications for further adaptation to OROV.

## Author summary

OROV is a midge-borne disease, and some field and experimental studies have evidence that mosquitoes may also be involved in OROV transmission. Previous vector studies have focused on historical OROV strains or vectors from non-endemic regions. At present, the current role of mosquitoes from endemic areas in the spread of contemporary OROV strains associated with the emergence since 2023 remains understudied. We reported a vector competence study of *Ae. albopictus* and *Cx. quinquefasciatus* for OROV from OROV-endemic (Brazil) and non-endemic (USA) areas and directly compared historical and contemporary OROV strains across multiple infection doses, including those that closely recapitulate human viremia levels. Our data allowed us to identify the higher infectivity of the OROV strain 240023 (contemporary) in *Ae. albopictus* bodies at both low and high doses. Our data showed that peridomestic or urban mosquitoes (*Ae. albopictus* and *Cx. quinquefasciatus*) exhibit very limited competence for OROV transmission. Our results indicate that the recent expansion of OROV into new geographic areas is unlikely to be driven by a shift from midges to these mosquitoes. These findings reinforce the need to prioritize control of *Culicoides paraensis*, which is the primary OROV vector.

## Introduction

Oropouche virus (OROV) is a neglected arbovirus that has caused Oropouche fever in the Amazon Basin since the 1950s [1,2]. Oropouche fever is usually characterized by self-limited febrile illness, but in some cases, it leads to neurological diseases, pregnancy complications, and death [1,3]. Currently, OROV has been estimated to cause more than 9.4 million infections in Latin America and the Caribbean [4]. No specific antivirals or vaccines are available to treat or prevent Oropouche fever.

Since November 2023, OROV has reemerged in Brazil, Bolivia, Peru, Colombia, Venezuela, and Ecuador, and has established local transmission in the Caribbean, including Cuba, Panama, Barbados, the Cayman Islands, and the Dominican Republic [5–7]. Furthermore, several traveler-return cases have been reported in the United States of America (USA), Canada, Germany, Italy, and Spain [8,9]. This re-emergence has been linked to a novel OROV reassortant identified in the Amazon region of Brazil, which has subsequently spread to areas where OROV had not been previously documented [10,11]. *In vitro* experiments demonstrated that this OROV reassortant exhibits faster replication in multiple cell lines and the ability to evade neutralizing antibodies in individuals previously infected with OROV. Additionally, the reassortant strain was more infectious in *Culicoides sonorensis* biting midges and had a shorter extrinsic incubation period compared to the ancestral strain [12,13].

OROV is primarily transmitted to humans by *Culicoides paraenesis* midges in the human-amplified and enzootic cycles, and pale-throated sloths (*Bradypus tridactylus*) are potential enzootic amplifier hosts [14]. However, some studies have suggested the involvement of mosquito species in OROV transmission, including *Culex quinquefasciatus* [5,15]. Although previous studies have demonstrated low vector competence in mosquitoes, including *Cx. quinquefasciatus* and *Aedes albopictus* [13,16–19], it remains unclear whether there are replicative differences between the reassortant and prototype OROV strains in mosquitoes from regions with recent outbreaks. Therefore, we evaluated the vector competence of *Cx. quinquefasciatus* and *Ae. albopictus* populations from OROV-endemic and non-endemic regions to determine if the OROV reassortant strain exhibits enhanced replication compared to the OROV prototype strain.

## Methods

### Oropouche fever cases and viral load

Serum samples were collected from 24 patients with confirmed Oropouche fever in Ceará State, Brazil, between July and August 2024 [20]. All cases were confirmed by real-time RT-qPCR targeting OROV RNA [21] and matched by age, sex, and the interval between symptom onset and sample collection. OROV-positive serum samples were titrated by focus-forming assay as previously described [12]. The samples were serially diluted in an 8-fold series in DMEM (1% penicillin-streptomycin). 100 µL of each dilution was used to inoculate Vero CCL-81 cells seeded on a 96-well plate. Following an incubation for 1 hour at 37˚C (5% CO_2_), 125 µL of DMEM supplemented with 0.75% carboxymethylcellulose and 5% fetal bovine serum (FBS) was added to each well and the plates were incubated for 48 hours at 37˚C (5% CO_2_). Following incubation, the cells were fixed with 70 µL of 4% paraformaldehyde solution (PFA) and incubated for 1 hour at 4˚C. The PFA was then removed, and cells were washed with phosphate-buffered saline (PBS). The cells were then blocked for 30 minutes with 150 μL of blotto before being washed with Perm/Wash Buffer (PBS supplemented with 0·1% BSA and 0·1% Triton X-100) and incubated with the polyclonal anti-OROV antibody (Cat no. VR-1228AF, ATTC, USA). After a second wash with Perm/Wash, the monolayers were incubated with an anti-mouse IgG secondary antibody (Cat no. AP124P, Sigma-Aldrich, USA). Finally, after a final wash with Perm/Wash Buffer, the foci were visualized using the True-Blue Peroxidase substrate (Cat no. 5510–0030, KPL, USA) for 30 minutes. Differences in viral load between females and males were assessed using the Welch Two Sample t-test in RStudio version 4·4·0.

### Mosquitoes

The colonies of *Cx. quinquefasciatus* and *Ae. albopictus* used in this study originated from a natural population sampled from Salvador, Brazil (Salvador line) or Houston, USA (MCOA line). The *Cx. quinquefasciatus* used in this study were from the 71^st^ generation of the Salvador line (F71), and *Ae. albopictus* were from the 28^th^ generation of both the Salvador and MCOA lines (F28). Mosquitoes were reared under standard insectary conditions consisting of 28ºC and 70 ± 10% relative humidity with a 12h:12h light-dark cycle. Eggs were hatched in deionized water, and larvae were fed fish food (TetraMin Tropical Flakes). Adults were held in BugDorm cages with constant access to 10% sucrose.

### Viruses

OROV strains BeAn 19991 and 240023 were acquired from the World Reference Center for Emerging Viruses and Arboviruses at the University of Texas Medical Branch, Galveston, USA. Lyophilized viruses were reconstituted in 0.5 mL of sterile water. Viral stocks were propagated in confluent flasks of Vero E6 cells using 100 µL of virus diluted in 900 µL of DMEM supplemented with 2% FBS and penicillin-streptomycin. Diluted inoculum was added to the cells and incubated for 1 hour at 37°C in 5% CO_2_. After incubation, 10 mL of DMEM supplemented with 2% FBS and 1% penicillin-streptomycin was added to the flasks, and OROV was cultured at 37°C with 5% CO_2_. After 30–36 hours, cell supernatant was collected and centrifuged at 1,734 g for 5 minutes to remove cell debris. Supernatant was aliquoted and stored at -80°C until use. Before use in mosquito infection assays, OROV strain 240023 was passaged four times in Vero cells, and OROV strain BeAn 19991 underwent three passages in suckling mouse brain followed by four passages in Vero cells.

### Vector competence assay

Five- to seven-day-old female mosquitoes were starved for 4 hours before the infectious blood meal was performed in an Arthropod Containment level 2 facility (ACL-2). *Cx. quinquefasciatus* and *Ae. albopictus* were experimentally exposed to either the BeAn 19991 or 240023 isolates at infectious doses of 4 (a low infectious dose within the range of human viremia) or 6 (a high infectious dose matching that used in previous studies) log_10_PFU/mL (S1 Table in S1 Data). Each OROV stock was diluted in DMEM plus 1·5% heat-inactivated FBS, 1% penicillin/streptomycin, and 1% antibiotic-antimycotic (Gibco, USA). One volume of virus suspension was mixed with two volumes of washed red blood cells originating from defibrinated sheep blood (Catalog number 31122, Colorado Serum Company, USA). A total of 2 mL of the infectious blood meal was added to Hemotek membrane feeders (Catalog number SP6W1-3, Hemotek, UK), which were covered with a piece of desalted porcine intestine as a membrane and maintained at 37°C. Mosquitoes were allowed to feed for 15 minutes in the dark. After feeding, fully engorged females were incubated in 0·5 liter cardboard cups under controlled conditions (28 ± 1°C; relative humidity, 75 ± 5%; 12h:12h light/dark cycle) in a climatic chamber. After 7, or 14 days of incubation, bodies, legs, and saliva were collected. For saliva collection, the mosquito proboscis was inserted into the tip of a 10 µL pipette pre-filled with FBS and allowed to salivate for 30 minutes. Then, 10 µL of FBS was added to 100 µL of cell culture medium and stored at -80°C prior to the plaque assay. Bodies and legs of OROV-exposed mosquitoes were stored in 2 mL screw-lid vials at -80ºC until they were processed.

### Plaque assay

To assess the mosquito infection rates of the different compartments (i.e., bodies and saliva), we performed a plaque assay on Vero E6 cells. Vero E6 cells were seeded into a 24-well plate and cultured overnight before infection. Either whole bodies or legs were homogenized individually in vials containing 0·1 mm of glass beads and 330 µL of DMEM with 1% penicillin-streptomycin, 1% antibiotic and antimycotic (Catalog number 15240062, Gibco, USA), and 1·5% heat-inactivated FBS in a Precellys homogenizer for 3 rounds of 20 seconds with a 30-second pause after every round. Samples were centrifuged following homogenization. For the bodies and legs, a volume of 100 µL of mosquito homogenate in 10-fold dilutions (neat to 10^5^) was added to cells then incubated at 37°C (5% CO_2_) for 1 hour, rocking every 15 minutes. The entire 100 µL saliva sample was used to inoculate the Vero E6 cells. The positive controls consisting of serial dilutions of the virus stock as well as negative controls consisting of uninfected media were also used to inoculate the Vero E6 cells. Infected cells were overlaid with OPTI-MEM media supplemented with 1·25% carboxymethyl cellulose, 5% FBS, and 1% P/S. After four days of incubation at 37°C, infected cells were fixed with 10% formalin for at least 1 hour and were washed with 1 × PBS and stained with crystal violet. This protocol was also used to back-titer the bloodmeals.

### Statistics

We performed a power analysis and determined that 12 patients per group provided 80% statistical power to detect a standardized mean difference (Cohen’s d = 1.20) of viral load of infectious OROV particles in serum samples between sex (females vs. males) at a two-sided significance level of α = 0.05. Significance between body infection, disseminated infection, and saliva infection rates of BeAN 19991 vs. 240023 within each experimental group as well as between the Salvador and MCOA lines was determined by two-tailed Chi-squared analysis. Viral titers among infected individuals was determined by Mann-Whitney U-test. Both were corrected for multiple comparisons using the Holm-Šídák method. A p-value < 0.05 was determined to denote a significant difference in values between experimental groups.

Given the variability between replicates, we performed a two-way ANOVA on the linear regression of the body infection rates to test the effect of replicate, virus strain, and the interaction between the replicate and virus strain on infection. As expected, for the *Ae. albopictus* 7 days post-infection (dpi), we see an effect of the interaction between the replicate and virus strain on body infection rates at both infectious doses, which is corroborated by lack of significance in the Chi-squared analysis. At 14 dpi in the high dose, we see a significant effect of replicate and virus strain on body infection rates, but importantly the interaction between replicate and virus strain is not significant, indicating the effect of the virus strain is consistent across replicates and the observed variability is due to differences in the magnitude of infection between replicates. The low dose shows a significant effect of the interaction between the replicate and virus strain, likely driven by the fact that in one replicate we saw zero infection with the BeAN 19991 strain, and 29% infection in the second replicate, but in both replicates the infection rate is higher in those exposed the 240023 strain (S2 Table in S1 Data and S1 Fig).

## Results

To assess the viremia during OROV infection in humans, we measured the viral load in serum samples collected from patients with Oropouche fever in Brazil within four days of symptom onset. To control for confounding variables, we matched patients by sex, age, and the interval between symptom onset and sample collection. We found that Oropouche fever patients had a median viral load of 3.9 log_10_(focus-forming units) (FFU/ml) with an interquartile range from 3.6 to 4.6 log_10_FFU/mL. No significant differences in viral load were observed between male and female patients, but the viral load slightly decreased from day 0 to day 4 post-symptom onset (Fig 1A-1B).

**Fig 1:**
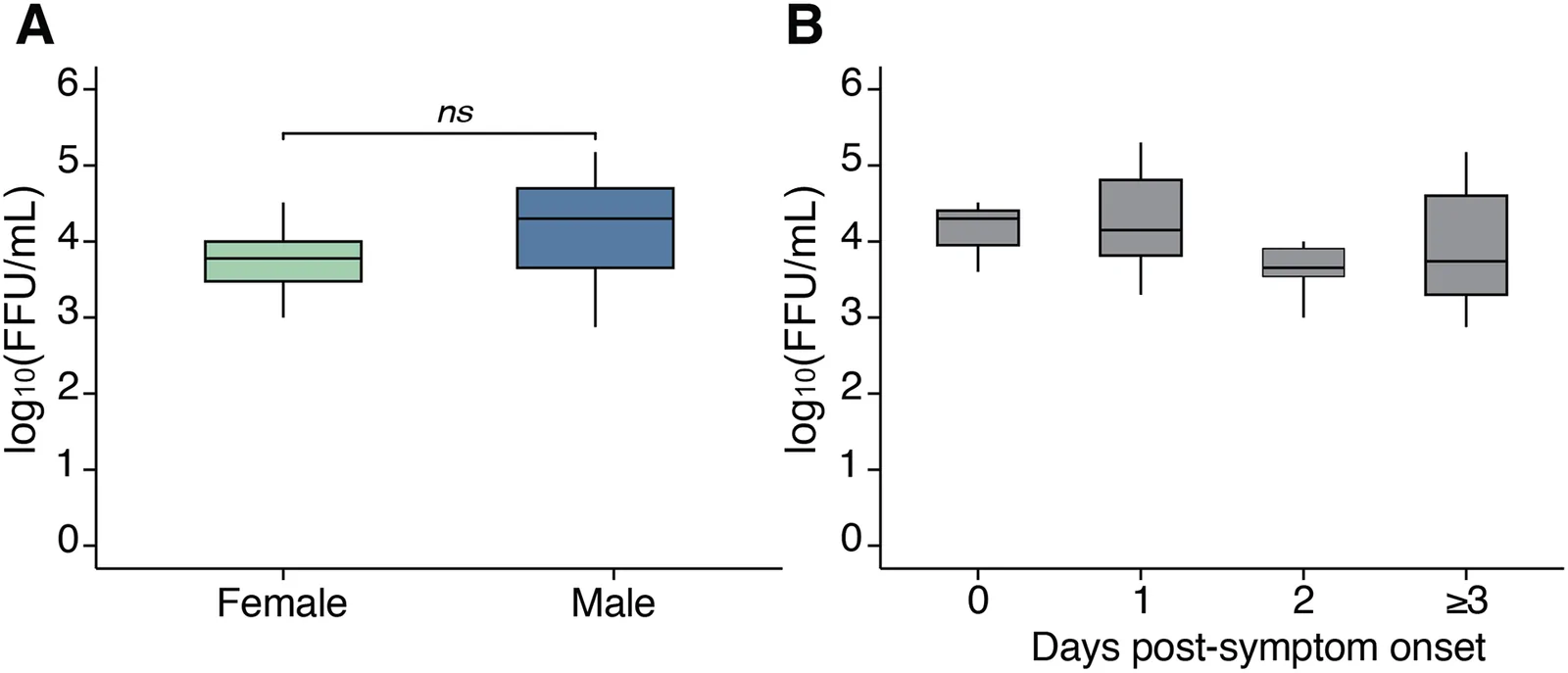
Viral load in Oropouche fever patients. A) Viral load of infectious OROV particles in serum samples from 24 Oropouche fever patients. Data are presented for female (n = 12) and male (n = 12) patients. B) Daily viral load of OROV in serum samples from 24 Oropouche fever patients following symptom onset. The slight decrease in viremia over time is not statistically significant. The box represents the interquartile range, and the whiskers extend to the minimum and maximum values. Significance was determined by Welch’s two-sample t-test.

To evaluate the vector competence of *Cx. quinquefasciatus* for OROV, a colony from Salvador, Brazil was exposed to either the BeAn 19991 strain (prototype isolated in 1960) or the 240023 strain (isolated in 2024) at two infectious doses: a low dose within the range of typical human viremia (3.6-4.6 log_10_PFU) and a high dose matching the dose used in prior studies (5 log_10_PFU). At 14 days post-infection, body, leg, and saliva samples were collected to assess body infection rates, disseminated infection rates, and saliva infection rates and viral titers, enabling comparison of infection dynamics and transmission potential between the two OROV strains. Body infection, disseminated infection, and saliva infection rates were calculated as the mean proportion of individual mosquitoes that contained infectious virus particles in the bodies, legs, and saliva, respectively, of those mosquitoes that imbibed an infectious bloodmeal.

At the low infectious dose, the BeAn 19991 strain showed significantly higher body infection rates than the 240023 strain (mean infection rate: 32·3% [N = 96] vs. 1.7% [N = 117]; p < 0·0001) (Fig 2A). However, at the high dose, body infection rates were low for both strains (1·5% for BeAn 19991 [N = 130] vs. 0% for 240023 [N = 114]; p = 0·1836), with no significant differences observed in disseminated infection rates (1·5% [N = 130] vs. 0.9% [N = 115]; p = 0·6347) or saliva infection rates (2·7% [N = 74] vs. 1·4% [N = 70]; p = 0·5926) (Fig 2B). Additionally, the mean infectious titer of the BeAn 19991 strain (x̄ = 2.08 log_10_PFU/mL; N = 31) was higher than that of the 240023 strain (x̄ = 1.15 log_10_PFU/mL; N = 2) in the bodies at the low infectious dose (p-value = 0.0359), although the low sample number in those exposed to the 240023 strain may be convoluting this result. (Fig 2C). Due to the low number of positive samples, statistics could not be conducted for each group, obfuscating the effect of each treatment on viral titer. However, viral titers in the legs and saliva generally remained low at about 1 log_10_PFU/mL, which is at the limit of detection (1 log_10_PFU/mL) (Fig 2C and 2D). Among mosquitoes exposed to the low dose, disseminated infection rates (2·1% [N = 48] for the BeAn 19991 strain vs. 2·9% [N = 68] for the 240023 strain; p = 0·7743) and saliva infection rates (2·1% [N = 48] vs. 1·5% [N = 69]; p = 0·7931) did not differ significantly between strains (Fig 2A).

**Fig 2:**
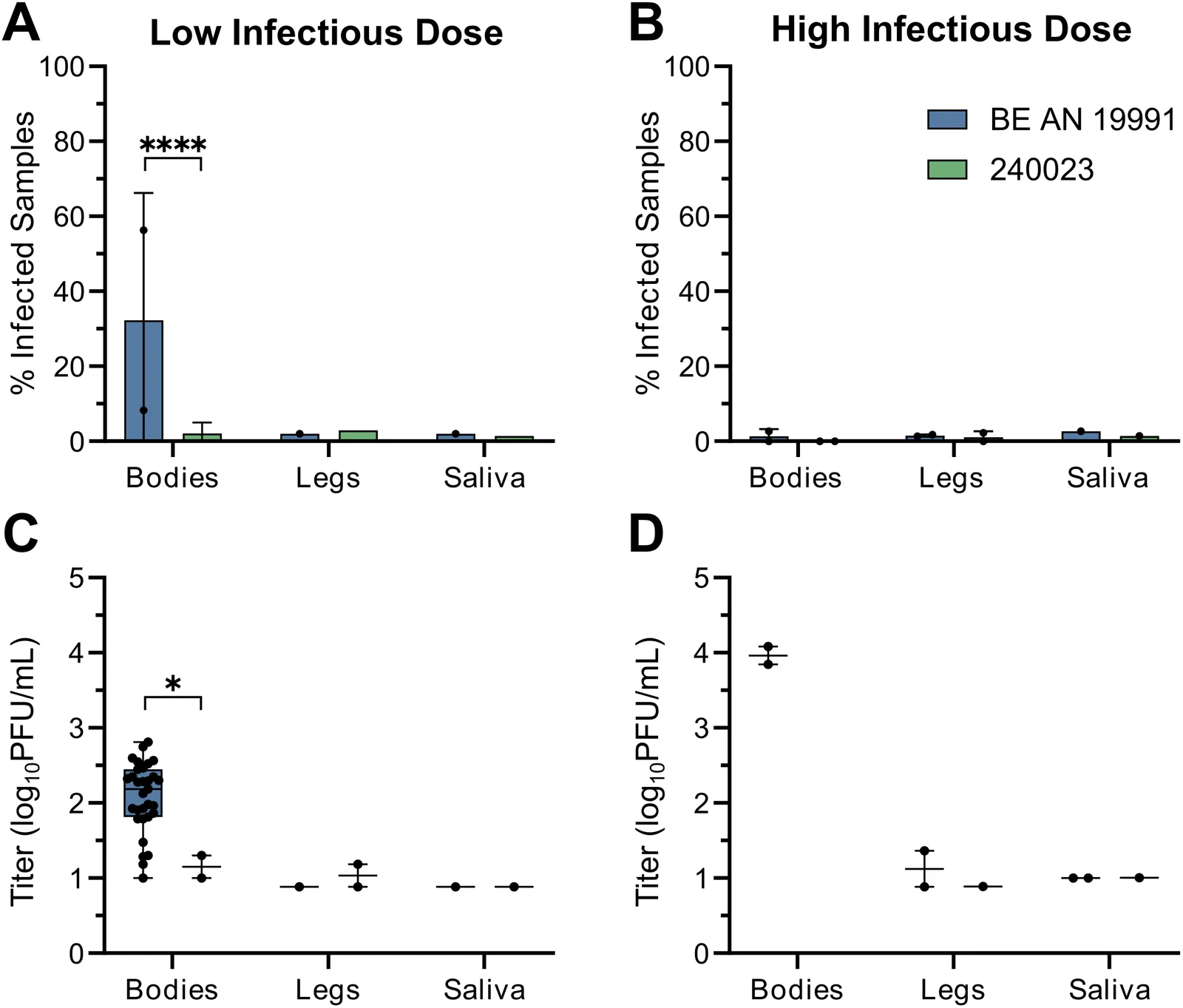
Vector competence of *Cx. quinquefasciatus* for OROV. OROV body infection, disseminated infection, and saliva infection rates in *Cx. quinquefasciatus* mosquitoes with BeAn 19991 or 240023 strains at a (A) low infectious dose (3.5 log10PFU) or (B) high infectious dose (5 log10PFU). Bodies, legs, and saliva were collected 14 days post-infection (dpi), and the presence of infectious viral particles was determined via plaque assay from 1-2 independent replicates. Data points represent the proportion infected in each replicate. Significance was determined using two-tailed Chi-squared analysis. Leg and saliva data represents the percent positive out of the total OROV challenged mosquitoes. Bar height represents the mean value of all replicates and error bars indicate standard deviation. Viral titers in the bodies, legs, and saliva at a (C) low or (D) high infectious dose were also determined and positive samples from all replicates were combined to determine the mean titer. Titers of individual samples are represented by a dot. The box represents the interquartile range, and the whiskers extend to the minimum and maximum values. Significance was determined by Mann-Whitney U test corrected for multiple comparisons using the Holm-Šídák method. Significant differences in infection rates between the BeAn 19991 or 240023 strains are marked as follows: ‘*’ p-value between 0.1 and 0.5, ‘****’ p-value < 0·0001.

To assess the vector competence of *Ae. albopictus* for OROV, a line of *Ae. albopictus* from Salvador, Brazil was challenged with the BeAn 19991 or 240023 strain at either a low infectious dose within the range of viremia of human hosts (3.6-4.6 log_10_PFU) or a high infectious dose matching the dose used in previous studies (6 log_10_PFU). Bodies, legs, and saliva were collected to assess body infection, disseminated infection, and saliva infection rates and viral titers, respectively, to compare the infection dynamics and transmission potential between both strains of OROV at 7 and 14 dpi. We found significantly higher body infection rates with the 240023 strain compared to the BeAn 19991 strain at both days 7 (BeAn 19991 x̄ = 3.3% [N = 150], 240023 x̄ = 15.4% [N = 156]; p-value = 0·0003) and 14 (BeAn 19991 x̄ = 8·6% [N = 105], 240023 x̄ = 29.6% [N = 125]; p-value < 0·0001) post infection in the high infectious dose (Fig 3B). In contrast, there was a significant increase in body infection rates for the 240023 strain at the low infectious dose 14 dpi (BeAn 19991 x̄ = 15·3% [N = 59], 240023 x̄ = 57.0% [N = 72]; p-value < 0·0001) but not 7 dpi (BeAn 19991 x̄ = 21.5% [N = 107], 240023 x̄ = 13.3% [N = 113]; p-value = 0·1069). There was a significantly higher saliva infection rate in the 240023 strain (x̄ = 6.4%, N = 125) compared to the BeAn 19991 strain (x̄ = 1.0%, N = 105) in the mosquitoes challenged with the high infectious dose 14 dpi (p-value = 0·0338) (Fig 3A). Disseminated infection and saliva infection rates remained below 10%, indicating that both strains exhibited limited transmission potential. There were no significant differences in viral titers between the BeAn 19991 and 240023 strains (Fig 3C-3D).

**Fig 3:**
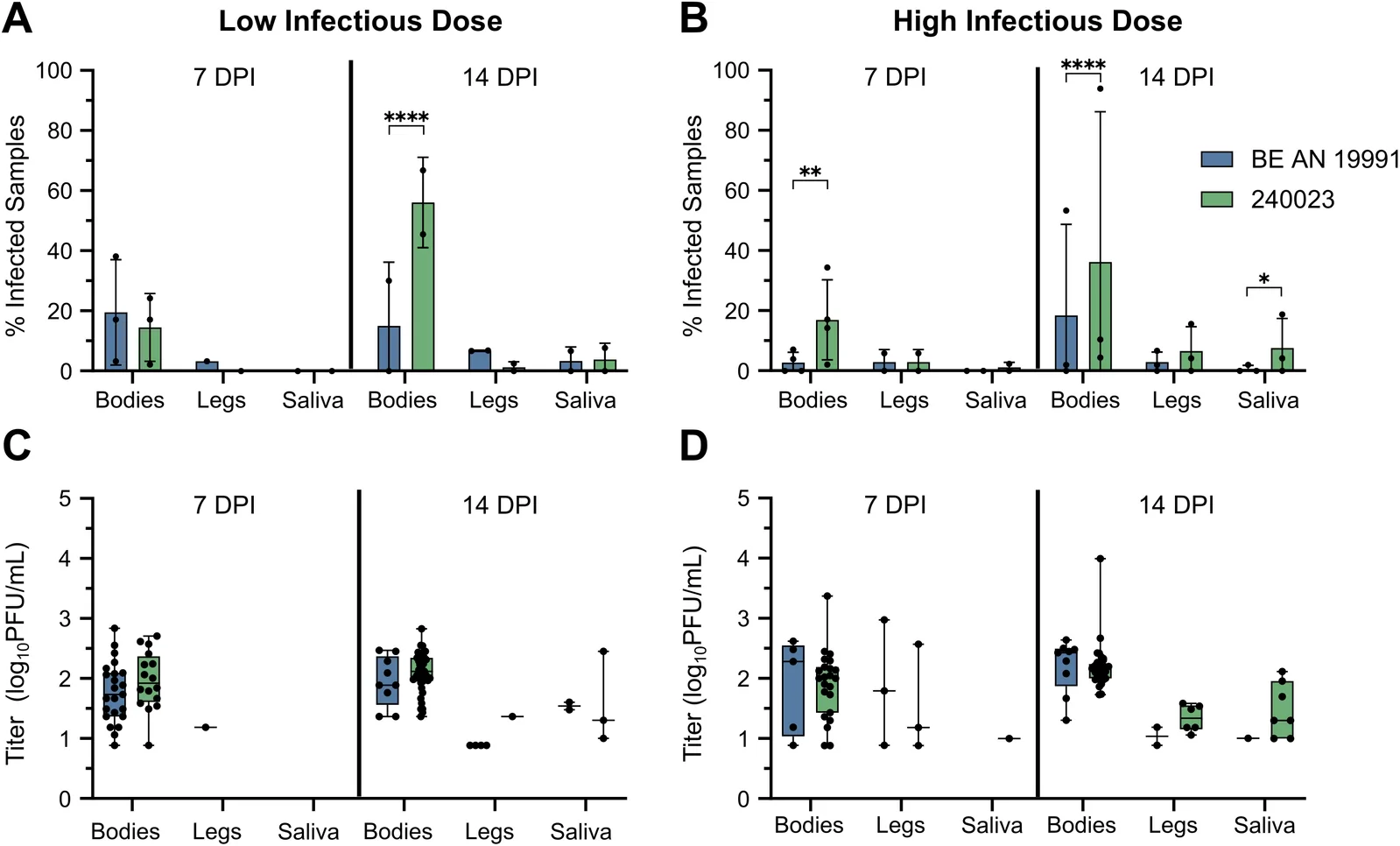
Vector competence of *Ae. albopictus* for OROV. OROV body infection, disseminated infection, and saliva infection rates of *Ae. albopictus* mosquitoes (Salvador line) challenged with BeAn 19991 or 240023 strains (**A**) low infectious dose (4 log_10_PFU) or (**B**) high infectious dose (6 log_10_PFU). Bodies, legs, and saliva were collected 7 and 14 dpi, and the presence of infectious viral particles was determined via plaque assay from 2-4 independent replicates. Data points represent the proportion infected in each replicate, and significant differences in infection rates between the BeAn 19991 and 240023 strains are marked as follows: ‘*’ p-value between 0.01 and 0.05; ‘**’ p-value between 0·001 and 0·1; ‘****’ p-value < 0·0001. Significance was determined via two-tailed Chi-squared analysis. Leg and saliva data represent the percent positive out of the total OROV-challenged mosquitoes. Bar height represents the mean value of all replicates and error bars indicate standard deviation. Viral titers in the bodies, legs, and saliva at a (**C**) low or (**D**) high infectious dose were also determined and positive samples from all replicates were combined to determine the mean titer. Titers of individual samples are represented by a dot. The box represents the interquartile range, and the whiskers extend to the minimum and maximum values. Significance was determined by Mann-Whitney U test corrected for multiple comparisons using the Holm-Šídák method.

Previous vector competence studies of the OROV strain 240023 in *Ae. albopictus* relied on mosquito lines from the USA [18]. To determine whether our findings from the Salvador City (Brazil) line were consistent across populations, we simultaneously tested a USA line from Houston, Texas (MCOA, F28). At 7 dpi with the low dose, both lines showed higher body infection rates with the BeAn 19991 strain (Salvador: 17·1% [N = 35] vs. 2·2% [N = 45], p = 0·0191; MCOA: 4·6% [N = 44] vs. 0% [N = 42], p = 0·1621). However, this result was not reproducible across experimental replicates (see Fig 3A). However, by 14 dpi, this trend reversed in the Salvador line, which showed significantly higher body infection rates with the 240023 strain (66·7% [N = 39] vs. 30·0% [N = 30], p = 0.0025), whereas the MCOA line showed no significant difference between strains (25·0% [N = 32] vs. 20·0% [N = 30], p = 0·6380). Notably, only the Salvador mosquito line showed evidence of transmission potential at the low dose, with 6·7–7·7% of mosquitoes having detectable OROV in saliva. No virus was detected in saliva from the MCOA line (Fig 4A). Following high-dose exposure, body infection rates increased in the Salvador line for the 240023 strain (0·0% [N = 45] vs. 34·4% [N = 32], p < 0·0001) at 7 dpi, though no such increase was observed in the MCOA line (13·6% [N = 44] vs. 3·0% [N = 33], p = 0·1091). By 14 dpi, both lines exhibited significantly higher body infection rates with the 240023 strain (Salvador: 93·8% [N = 32] vs. 53·3% [N = 15], p = 0·0010; MCOA: 96·9% [N = 32] vs. 3·3% [N = 30], p < 0·0001). In both mosquito lines, disseminated infection rates remained low across strains. However, only the Salvador line showed viral presence in saliva across all infectious doses (Fig 4A and 4B). At the low infectious dose, saliva infection rates were low and not significantly different between OROV strains (6·7% [N = 30] vs. 7·7% [N = 39], p = 0·8706), but following exposure to the high infectious dose, 18·8% of mosquitoes fed the 240023 strain had infectious virus in their saliva (N = 32), but those fed the BeAn 19991 strain did not (N = 15) (Fig 4B).

**Fig 4:**
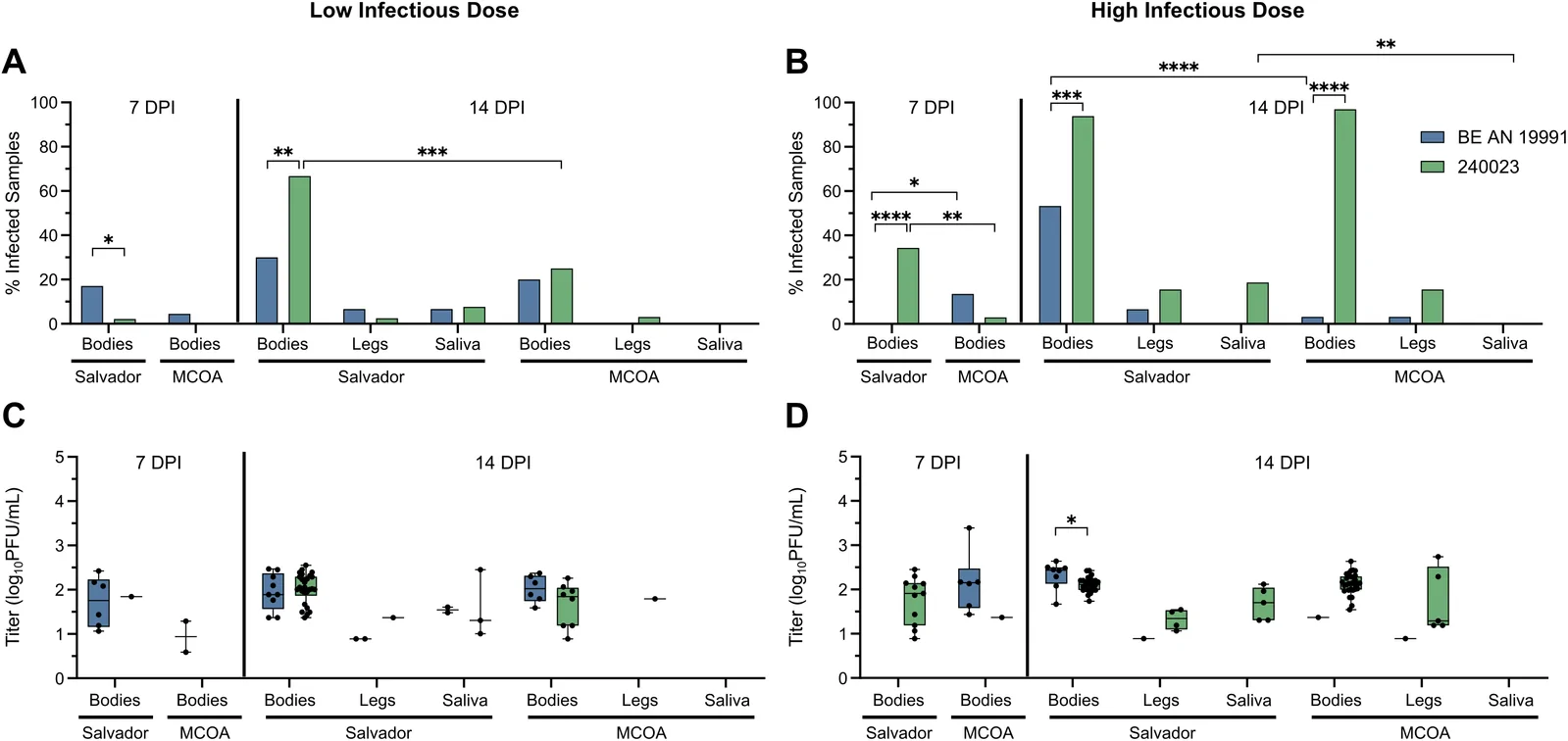
Transmission potential of *Ae. albopictus* for OROV by mosquito line. OROV infection rates in two unrelated lines of *Ae. albopictus* from Salvador City, Brazil (Salvador) and Houston, USA (MCOA) with BeAn 19991 or 240023 strains at a (**A**) low infectious dose (4 log_10_PFU) or (**B**) high infectious dose (6 log_10_PFU). Whole bodies were collected 7 dpi, and bodies, legs, and saliva were collected 14 dpi. The body infection rate was determined via plaque assay. Significance was determined via two-tailed Chi-squared analysis. Viral titers in the bodies, legs, and saliva at a (**C**) low or (**D**) high infectious dose were also determined by plaque assay and the titer of each individual sample is represented by a dot. The box represents the interquartile range, and the whiskers extend to the minimum and maximum values. Significance was determined by Mann-Whitney U test corrected for multiple comparisons using the Holm-Šídák method. Significant differences in infection rates and titers between the BeAn 19991 and 240023 strains as well as mosquito lines are marked as follows: ‘*’ p-value between 0.01 and 0.05; ‘**’ p-value between 0.001 and 0.1; ‘****’ p-value < 0.0001.

There were significant differences in body infection and saliva infection rates between the Salvador and MCOA lines. At 7 dpi, the BeAn 19991 strain had a higher body infection rate in the MCOA line (x̄ = 0% [N = 45] vs. 13.6% [N = 44]; p-value = 0.01) while the 240023 strain had a higher body infection rate in the Salvador line (x̄ = 34.4% [N = 32] vs. 3.0% [N = 33]; p-value = 0.0011) at the high infectious dose. Similarly, at 14 dpi BeAn 19991 had a higher body infection in the Salvador line compared to the MCOA line (x̄ = 53.3% [N = 15] vs. 3.3% [N = 30]; p-value < 0.0001) but the 240023 strain infects both lines at similar levels. However, saliva infection rates of the 240023 strain were higher in the Salvador line (x̄ = 18.8% [N = 32] vs. 0% [N = 32]; p-value = 0.0101) compared to the MCOA line (Fig 4B). At the low infectious dose, only the 240023 strain exhibited significantly different infection rates, having increased body infection rates in the Salvador line compared to the MCOA line 14 dpi (x̄ = 66.7% [N = 39] vs. 25.0% [N = 32]; p-value = 0.0005) (Fig 4A). Viral replication was similar between BeAn 19991 and 240023 except in the bodies at the high infectious dose 14 dpi (x̄ = 2.20 log_10_PFU/mL [N = 9] vs. 2.19 log_10_PFU/mL [N = 30]; p-value = 0.0138) (Fig 4C-4D). All results and statistics are summarized in S3 Table in S1 Data.

## Discussion

This study demonstrates that *Cx. quinquefasciatus* and *Ae. albopictus* have low vector competence for the contemporary OROV reassortant strain at inoculation doses equal to and greater than peak viremia in patients, as evidenced by the low percentage of mosquitoes with infectious saliva. This suggests that the putative increases in infection rates in mosquitoes associated with the contemporary reassortant strain may not significantly impact its ability to be transmitted by *Cx. quinquefasciatus* and *Ae. albopictus* in peri-urban environments. This refractoriness aligns with current epidemiological evidence that OROV circulates primarily within human populations in rural areas, strongly supporting the conclusion that midges remain the primary OROV vectors [22,23]. Our study corroborates previous studies that evaluated historical OROV strain isolates, which also demonstrated limited OROV replication in the midgut of *Ae. aegypti*, *Ae. albopictus*, and *Cx. quinquefasciatus* [17,24,25]. Additionally, our findings aligned with experimental infections using *Cx. quinquefasciatus* and *Ae. albopictus* from the United States, which also demonstrated a general lack of competence for historic and contemporary OROV strains [18]. We hypothesize that OROV detection in *Cx. quinquefasciatus* and *Ae. albopictus* might be attributed to incidental infections or occur at very low rates, potentially influenced by genetic variations within *Cx. quinquefasciatus* and *Ae. albopictus* populations. Alternatively, OROV detection in mosquito species may reflect residual infectious blood in the midguts of exposed but uninfected mosquitoes tested.

Our results highlight the importance of using geographic and genetic backgrounds of mosquitoes paired with relevant viral genotypes or strains when performing vector competence assays. The increased infectivity of the reassortant 240023 strain was consistent across the two genetic backgrounds of *Ae. albopictus* evaluated in this study, but detection of the OROV in the saliva was dependent on the *Ae. albopictus* genetic background, with none of the mosquito individuals from a North American line developing infectious saliva compared to up to 20% of the Salvador line. Differences in arbovirus infection rates between different lines of mosquitoes have been reported on numerous occasions, highlighting that vector competence is a quantitative genetic trait in mosquitoes [26–34]. Further research is necessary to determine whether these putative geographic variations in susceptibility reflect mosquito genetics or other intrinsic factors, such as the microbiome, virome, or innate immune response of these mosquito populations. We acknowledge that a limitation of this study is that *Ae. albopictus* have been maintained in laboratory settings for multiple generations and may not fully reflect the genetic background of the wild populations.

Our data also demonstrates the importance of vector competence studies that use bloodmeals mimicking human viremia and testing a range of infectious doses before drawing conclusions about mosquito-virus pairings. Our study shows that the bodies of Ae. albopictus can be efficiently infected with OROV even at low oral doses, although disseminated infections and transmission potential remain limited. Interestingly, we did not observe a dose-dependent increase in infection. Differences in the magnitude of immune responses across OROV doses could explain the absence of a dose-dependent infection we observed. Further work is needed to clarify this.

A previous study found that Ae. aegypti was refractory to OROV infection when exposed through an infectious bloodmeal, but 100% of mosquitoes were infected following intrathoracic injection, suggesting the presence of a midgut infection or escape barrier [19]. The infection rates observed in Ae. albopictus bodies suggest that OROV can overcome the midgut infection barrier but low disseminated and saliva infection rates show that the virus fails to disseminate into the hemocoel, indicating a possible midgut escape barrier, but this remains to be tested. The contemporary. As with most mosquito-virus systems, Ae. aegypti has been shown to mount an immune response to OROV^19^. We hypothesize that differences in body infection rates between the two OROV strains may be due to differences in their ability to activate or evade the mosquito immune response.

In conclusion, our study demonstrates that *Cx. quinquefasciatus* and *Ae. albopictus* are relatively refractory to infection with recently emerged OROV strains. This suggests that *Cx. quinquefasciatus* and *Ae. albopictus* likely do not play a significant role in the OROV transmission cycle. These findings strongly emphasize the importance of continued vector control efforts targeting midges as a primary approach to effectively mitigate and interrupt OROV transmission.

## Supporting information

S1 Fig Body infection rates of mosquitoes by experimental replicate. OROV mean body infection rates of *Cx. quinquefasciatus* (14 dpi [A]) and *Ae. albopictus* (7 dpi [B] and 14 dpi [C]) mosquitoes challenged with two doses of either the BeAn 19991 or 240023 strains. Significance was determined via two-tailed Chi-squared analysis. Significant differences in infection rates and titers between the BeAn 19991 and 240023 strains as well as mosquito lines are marked as follows: ‘*’ p-value between 0.01 and 0.05; ‘**’ p-value between 0.001 and 0.1; ‘***’ p-value between 0.001 and 0.0001; ‘****’ p-value < 0.0001.
(TIFF)

S1 Data S1 Table. OROV Bloodmeal titers by replicate. S2 Table. Analysis of deviance tables for body infection rates. S3 Table. Body infection, disseminated infection, and saliva infection rates and titers.
(XLSX)
